# Enzalutamide Prolonged the Duration of Drug Use in Comparison to Abiraterone Acetate and Cabazitaxel after Upfront Docetaxel: A Large Japanese Database Study

**DOI:** 10.3390/diseases12070162

**Published:** 2024-07-18

**Authors:** Katsuya Yamaguchi, Takashi Kawahara, Akihito Hashizume, Kimito Ousaka, Koichi Uemura, Yusuke Ito, Hiroki Ito, Kazuhide Makiyama, Hiroji Uemura

**Affiliations:** 1 Departments of Urology and Renal Transplantation, Yokohama City University Medical Center, Yokohama 2320024, Japan; katsuya333@gmail.com (K.Y.); pjcbn002@yahoo.co.jp (A.H.); ousaka@yokohama-cu.ac.jp (K.O.); hu0428@med.yokohama-cu.ac.jp (H.U.); 2 Department of Urology, Yokohama City University, Graduate School of Medicine, Yokohama 2360004, Japan; uemura.koi.pf@yokohama-cu.ac.jp (K.U.); yitou1@yokohama-cu.ac.jp (Y.I.); hiroki22@yokohama-cu.ac.jp (H.I.); makiya@yokohama-cu.ac.jp (K.M.)

**Keywords:** docetaxel, upfront, metastatic, CRPC, CSPC

## Abstract

Introduction: In the United States, a total of 268,490 men were found to have prostate cancer in 2022, thus making it the most common cancer in men, accounting for 27% of all cancers in the male population. Among all cancers in men, it was the fifth leading cause of death, with 34,500 deaths and a mortality rate of 11%. In 2019, the total number of cases was 94,748, making it the leading cancer in males, accounting for 11% of all male cancers. In terms of mortality, it ranked seventh, with 13,217 deaths and a mortality rate of 1.6%. However, new treatment options for metastatic castration-sensitive prostate cancer (mCSPC) have emerged. Docetaxel has been shown to be effective for both mCSPC and castration-resistant prostate cancer (CRPC). Upfront docetaxel has not been approved in Japan, nor has it been validated in large-scale studies. Furthermore, several agents can be used after docetaxel treatment, but it is unclear which is the most effective. We used a large Japanese health insurance database to determine which agent would be the most effective as a next-line therapy in patients who had received docetaxel. Materials and Methods: We used data from medical institutions using the Diagnosis Procedure Combination (DPC), which provides a comprehensive evaluation of medical classifications. The Medical Data Vision database covers approximately 23% of DPC hospitals in Japan. This study analyzed 2938 patients with mCSPC who received docetaxel, followed by CRPC, between April 2008 and December 2021. The study focused on three agents: enzalutamide, abiraterone acetate, and cabazitaxel. Other agents were excluded due to the small number of patients. The following data were analyzed: age, date of CRPC diagnosis, presence of bone metastasis, drug type, and prognosis. Results: This study included 1997 patients with CRPC after upfront docetaxel therapy for mCSPC (enzalutamide [ENZ] group, n = 998; abiraterone acetate [ABI] group, n = 617; and cabazitaxel [CBZ] group, n = 382). The overall survival (OS) time from drug initiation was 456 days in the enzalutamide group, which was significantly longer than that in the cabazitaxel group (*p* = 0.017, HR 0.94) (ENZ: ABI *p* = 0.54, HR 0.94; ABI: CBZ *p* = 0.14, HR 0.75). OS was also compared for the third-line drug in the group that received enzalutamide as the second-line drug, the group that used abiraterone acetate as the third-line drug (ENZ-ABI group), and the group that used abiraterone acetate as the second-line drug. OS from the start of the third-line drug was compared between the ENZ–ABI group and the ABI–ENZ group, which received enzalutamide as the third-line drug, but showed no significant difference (269 vs. 281 days, *p* = 0.85; HR 1.03). Conclusion: ENZ was shown to prolong OS relative to cabazitaxel after the cessation of docetaxel. ENZ was associated with a longer duration of drug use than ABI and CBZ.

## 1. Introduction

In 2022, prostate cancer was the most common cancer in men in the United States, accounting for 27% of all cancers, and affecting 268,490 men. In terms of mortality, it ranked fifth among all cancers in men, with 34,500 deaths and a mortality rate of 11% [1,2]. In 2019, a total of 94,748 people in Japan were reported to be affected, accounting for 11% of all cancers in men, making it the most common cancer in the male population. In the same year, 13,217 deaths were attributed to prostate cancer, which is a mortality rate of 1.6%, ranking it seventh among all male cancers [3].

New treatment options for metastatic castration-sensitive prostate cancer (mCSPC) have emerged in recent years [3]. Docetaxel has demonstrated effectiveness in hormone-sensitive prostate cancer with distant metastases in the CHAARTED and STAMPEDE trials [4,5], in addition to its use in the treatment of castration-resistant prostate cancer (CRPC).

Docetaxel (DOC) is a second-generation taxane drug extracted from the leaves of European yew trees. The main mechanism of action of taxanes is binding to microtubules and inhibiting cell division by promoting and stabilizing microtubule polymerization [6]. In addition to DOC, paclitaxel is another taxane drug. DOC shares a microtubule-binding site with paclitaxel, but DOC is thought to have a higher affinity and microtubule-stabilizing activity [7]. DOC also induces apoptosis [8,9] and inhibits angiogenesis. The TAX327 trial showed that DOC was more effective against prostate cancer than mitoxantrone in patients with mCRPC. A total of 1006 patients with mCRPC received DOC (75 mg/m^2^, every 3 weeks) or mitoxantrone (12 mg/m^2^, every 3 weeks). The TAX327 trial compared 1006 patients with mCRPC who received DOC (75 mg, every 3 weeks) and mitoxantrone (12 mg, every 3 weeks). Results showed a significant increase in OS in the DOC group (18.9 months vs. 16.5 months in the mitoxantrone group) (HR = 0.76) [5].

The CHAARTED trial evaluated 790 mCSPC patients randomized to receive androgen deprivation therapy (ADT) alone or six courses of DOC. The results showed a significant increase in overall survival (OS) in the ADT + DOC group (57 and 44 months) in comparison to the ADT alone group (HR = 0.61) [4]. The STAMPEDE study randomized 1081 mCSPC patients to ADT + DOC or ADT alone, and demonstrated that OS was significantly prolonged in the ADT + DOC group (81 and 71 months) in comparison to the ADT alone group (HR = 0.76) [10]. The DOC and abiraterone acetate groups were also compared, and no significant difference in OS was observed between the two groups, leading to the conclusion that upfront DOC is as effective as abiraterone acetate [5]. In contrast, the GETUG-AFU15 trial showed no significant difference in the OS of patients who received upfront DOC [11]. However, the randomized controlled trials of the CHAARTED and GETUG studies demonstrated an improved survival in DOC patients with high-volume metastases [12].

These studies have demonstrated the efficacy of upfront DOC for mCSPC treatment. The 2019 NCCN guidelines recommended six courses of Docetaxel (75 mg/m^2^) as first-line treatment for mCSPC. However, in the 2023 NCCN guidelines, the use of upfront DOC alone was removed and ADT + DOC + abiraterone or darolutamide triplet therapy was added instead [2]. This is because data from the PEACE-1 and ARASENS trials showed that ADT with DOC and triple therapy with abiraterone or darolutamide improved OS relative to ADT with DOC [13].

With the economic advantage of chemotherapy being that it is less expensive than androgen receptor signaling inhibitor (ARSI) treatment, DOC is expected to continue to be an important therapeutic option in the treatment of prostate cancer. In addition, there are a certain number of patients in whom the treatment response to upfront DOC according to the 2019 NCCN recommendations has remained stable.

Upfront docetaxel has not been approved in Japan, nor has it been validated in large-scale studies. Furthermore, several agents can be used after docetaxel treatment, but it is unclear which is the most effective [14]. We used a large Japanese health insurance database to determine which agent would be most effective as the next-line therapy after upfront DOC.

It is still unclear which drug should be used after upfront DOC. This study compared the OS of patients with CRPC after upfront DOC, divided into the ENZ, ABI, and CBZ groups, according to the second drug after DOC. ENZ is a small molecule androgen receptor inhibitor. ABI is an inhibitor of cytochrome P-450c17, an enzyme that is important in androgen synthesis. CBZ is a second-generation taxane that inhibits microtubule dynamics more potently than DOC, and has excellent intracellular retention. We investigated which of these (ENZ, ABI, or CBZ) was the optimal drug for use after upfront DOC (X1,X2,X3).

## 2. Methods

In this study, we used data from medical institutions using the Diagnosis Procedure Combination (DPC) system, which is a comprehensive reimbursement system for acute inpatient care in Japan. In April 2022, 1764 hospitals in Japan adopted the DPC system. In this study, DPC data were obtained from Medical Data Vision (Tokyo, Japan), whose database covers 23% of DPC hospitals in Japan [1].

**Figure 1:**
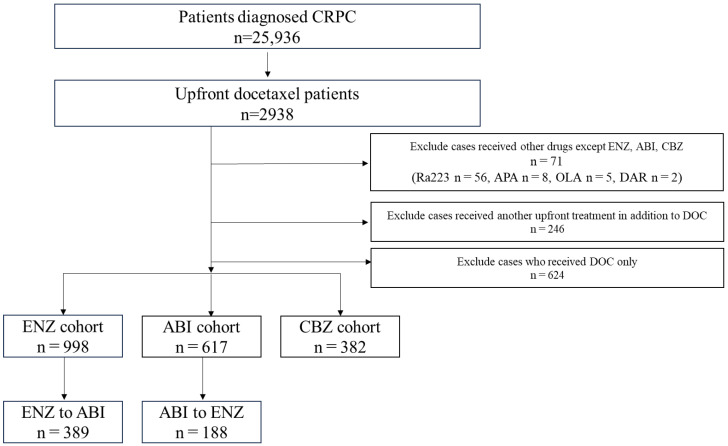
Patient selection. Of the 2938 patients who received upfront docetaxel (DOC), those excluded from the analysis were 71 patients who used Radium 223, apalutamide, olaparib, or darolutamide; 246 patients who received upfront treatment in addition to DOC; and 624 patients who received only DOC treatment. Thus, 1997 patients were included in the analysis (enzalutamide group, n = 998; abiraterone acetate group, n = 617; cabazitaxel group, n = 382).

From April 2008 to December 2021, patients diagnosed with prostate cancer were selected using the corresponding ICD-10 code (diagnosis code C61). This study included 2938 patients with mCSPC who received DOC and subsequently developed CRPC. Among these patients, 71 who used drugs other than ENZ, ABI, or CBZ after DOC were excluded because of the limited number of cases. We also excluded 246 patients who used drugs other than DOC at the mCSPC stage and 624 who used only DOC during the course of treatment. The drugs administered after upfront DOC included ENZ (n = 998), ABI (n = 617), and CBZ (n = 382) (Figure 1).

### Statistical Analyses

Both age and the duration of treatment were assessed using the Mann-Whitney U test. OS was expressed as Kaplan-Meier curves, and the log-rank test was used for comparisons. Tests were performed using the Graph Pad Prism software program (Graph Pad Software, Version 9.5.1, La Jolla, CA, USA). *p* values of <0.05 were considered to indicate statistical significance.

## 3. Results

**Figure 2:**
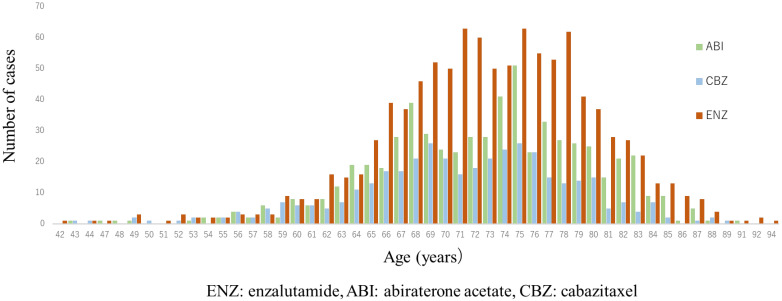
Age distribution of patients in 2nd line treatment (enzalutamide, abiraterone, and cabazitaxel group). Median age was both 73 years in enzalutamide (ENZ) and abiraterone acetate (ABI) groups, and 72 years in cabazitaxel (CBZ) group; CBZ group was younger than ENZ and ABI groups. (ENZ vs. ABI: *p* = 0.50, ENZ vs. CBZ: *p* < 0.0001, ABI vs. CBZ: *p* = 0.001).

**Figure 3:**
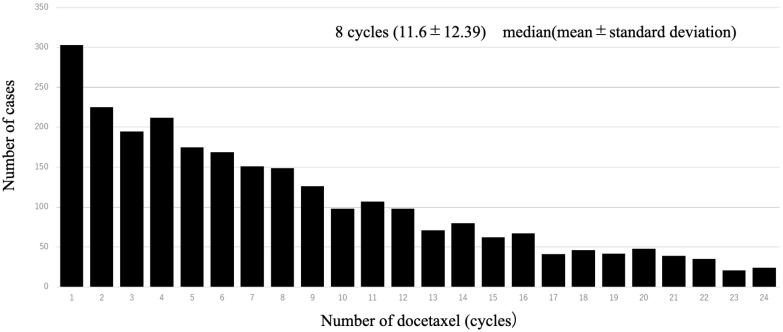
Number of patients per dose of upfront docetaxel. The table shows the number of each patient who received upfront docetaxel; the most common number of doses of upfront docetaxel was 1.

A total of 1997 cases were analyzed in this study. According to the drugs that they received after DOC, they were categorized as follows: included ENZ (n = 998), ABI (n = 617), and CBZ (n = 382) (Figure 1). Table 1 shows the patient characteristics of each treatment group. The CBZ group was younger than the ENZ and ABI groups. The median age was 72 (71.1 ± 7.36) years in the CBZ group, 73 (72.9 ± 6.98) years in the ENZ group, and 73 (72.6 ± 7.09) years in the ABI group (Table 1) (Figure 2). The median number of DOC treatment cycles administered to patients with mCSPC was 8 (11.6 ± 12.39) (Figure 3).

**Figure 4:**
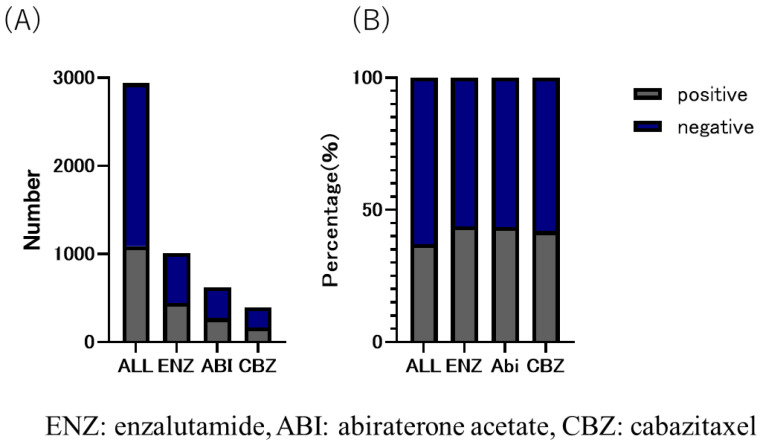
(**A**) number and (**B**) ratio of bone metastatic CRPC in enzalutamide, abiraterone acetate, and cabazitaxel group. The overall proportion of patients with bone metastases was 37.1%; 43.9% in eanzalutamide (ENZ) group, 43.5% in abiraterone acetate group, and 42.0% in cabazitaxel group, with the highest proportion in the ENZ group.

The CBZ group received the lowest number of DOC treatment cycles among the three groups. The number of treatment cycles were as follows: CBZ group, six (8.6 ± 8.39); ENZ group, eight (12.4 ± 13.23); and ABI group, eight (11.9 ± 12.71) (Table 1). The median time to CRPC was 180 days (495.9 ± 498.51) in the CBZ group, 420 days (583.59 ± 569.79) in the ABI group, and 510 days (692.3 ± 626.72) in the ENZ group (Table 1). The duration of use of the three drugs was as follows: CBZ group, 151 days (232 ± 238.41); ENZ group, 206 days (374.3 ± 453.56); and ABI group, 176 days (309.7 ± 351.10). The overall proportion of patients with bone metastasis was 37.1% (CBZ group, 42.0%; ENZ group, 43.9%; and ABI group (43.5%) (Figure 4).

**Figure 5:**
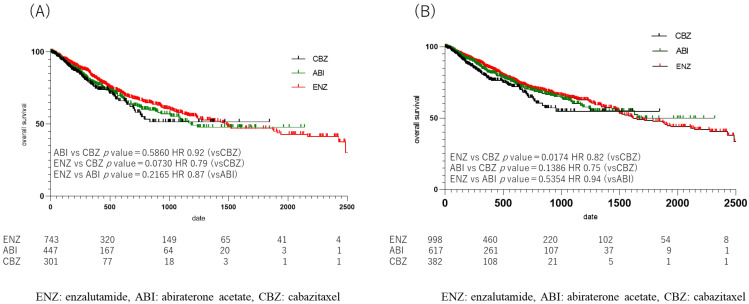
Kaplan Meier Curve in 2nd line treatment in enzalutamide, abiraterone acetate, and cabazitaxel group. (**A**) upfront docetaxel treatment less than one year, (**B**) all cases. (**A**) Median overall survival (OS) was shorter in the cabazitaxel (CBZ), abiraterone acetate (ABI), and enzalutamide (ENZ) groups, in that order, but there was no statistically significant difference when comparing OS of the 3 drugs (ENZ vs. ABI *p* = 0.22, HR 0.87, ABI vs. CBZ *p* = 0.59, HR 0.92, ENZ vs. CBZ *p* = 0.07, HR 0.79). (**B**) Comparison of overall OS in the ENZ, ABI, and CBZ groups showed a difference between the ENZ and CBZ groups (*p* = 0.017, HR 0.94).

The median OS from drug initiation in each group was 258 days (364.80 ± 472.77) in the CBZ group, 456 days (643.07 ± 595.13) in the ENZ group, and 390.5 days (539.90 ± 326.44) in the ABI group. A comparison of OS in each group showed a significant difference between the ENZ and CBZ groups (*p* = 0.017, HR 0.94) (Figure 5B); no significant difference in OS was observed between the ENZ and ABI groups (Figure S1), or between the ABI and CBZ groups (ENZ: ABI, *p* = 0.54; HR, 0.94; ABI: CBZ, *p* = 0. 14, HR 0.75). When the duration of upfront DOC use was limited to one year, the median OS was 246.5 days (352.37 ± 325.36) in the CBZ group, 405.5 days (604.39 ± 582.29) in the ENZ group, and 333.5 days (478.46 ± 430.83) in the ABI group, which was similar to the duration in the CBZ group. The duration of the ABI group was 333.5 days (478.46 ± 430.83). However, when the OS was compared among patients who received the three drugs, no statistically significant difference was observed (ENZ: ABI, *p* = 0.22; HR, 0.87; ABI: CBZ, *p* = 0.59; HR, 0.92; ENZ: CBZ, *p* = 0.07; HR, 0.79) (Figure 5A).

**Figure 6:**
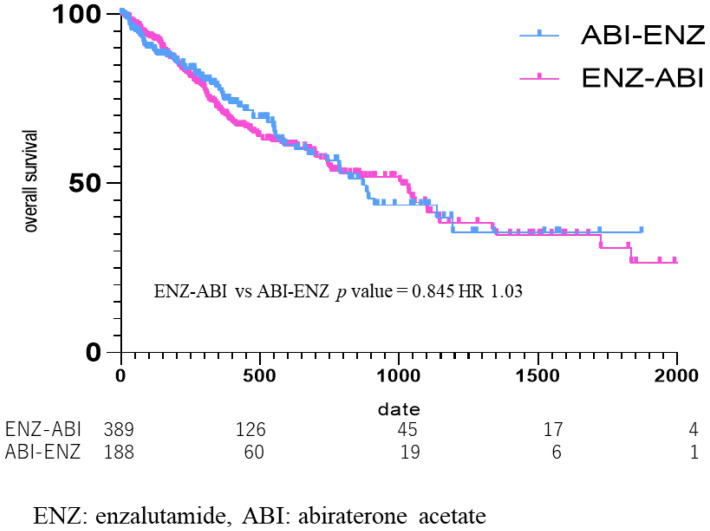
Kaplan Meier Curve in enzalutamide to abiraterone acetate group vs. abiraterone acetate to enzalutamide group. Median OS was 269 days in the enzalutamide (ENZ)–abiraterone acetate (ABI) group, and 281 days in the ABI–ENZ group, with no statistical difference in overall survival between the two groups (*p* = 0.85, HR 1.03).

OS was also compared according to the third-line drugs (Figure 6), from the start date of the third-line drug in the group that used ENZ as the second-line drug and ABI as the third-line drug (ENZ–ABI group, n = 395), and in the group that used ABI as the second-line and ENZ as the third-line drug (ABI–ENZ group, n = 193). The median OS from third-line treatment was 269 days (434.30 ± 425.36) in the ENZ–ABI group and 281 days (400.73 ± 385.11) in the ABI–ENZ group, which did not amount to a significant difference (ENZ–ABI vs. ABI–ENZ *p* = 0.85, HR 1.03) (Figure 6).

## 4. Discussion

This study included the largest number of patients in Japan who received upfront DOC. The sizes of the studies that analyzed upfront DOC were as follows: the CHAARTED study included 397 patients in the ADT + DOC group and 393 in the ADT alone group, out of a total of 790 patients [4]; the STAMPEDE study included 362 patients in the ADT + DOC group and 724 in the ADT alone group, out of 1081 patients [5]; the GETUG–AFU 15 study included 192 patients in the ADT + DOC group and 193 in the ADT alone group, out of a total of 385 patients [12]. In the GETUG–AFU 15 study, 192 and 193 patients were assigned to the ADT + DOC and ADT alone groups (total: 385 patients) [12]. We analyzed 2938 patients who received upfront DOC for mCSPC. The following drugs were used after DOC: ENZ (n = 998; 48.3%), ABI (n = 617; 29.8%), CBZ (n = 382; 18.5%), Ra-223 (n = 56; 2.7%), APA (n = 8; 0.39%), olaparib (n = 5; 0.24%), and DAR (n = 2; 0.1%). Based on the number of cases, 1997 patients who received ENZ, ABI, and CBZ were analyzed. Based on the above, the present study examined a large number of patients. The CHAARTED study included eight Asian patients [4], but the STAMPEDE and GETUG–AFU 15 studies did not include Asian patients. This is the first large-scale study of upfront DOC in the Japanese population.

In this study, the CBZ group did not reach the median survival time, but the ENZ group showed a significant prolongation of OS in comparison to the CBZ group (*p* = 0.017, HR 0.82), although there was no significant difference between the ENZ group and the ABI group, and between the ABI group and the CBZ group (ENZ vs. ABI: *p* = 0.54 HR 0.94 ABI vs. 0.94 ABI vs. CBZ: *p* = 0.14 HR 0.75) (Figure 5). ENZ was used longer than the other two drugs, and the proportion of patients with bone metastasis was highest in the ENZ group (Figure 4). Based on the above, ENZ is considered to be the second-best drug to use after upfront DOC. The comparison of the ENZ and CBZ groups in this study revealed a number of differences. First, the mean age of the ENZ group was 73 years (72.9 ± 6.98), while the mean age of the CBZ group was 72 years (71.1 ± 7.36), which was significantly different (*p* < 0.001). Second, the number of cycles of upfront DOC was eight (12.4 ± 13.23) in the ENZ group and six (8.6 ± 8.39) in the CBZ group (*p* < 0.0001). The time to CRPC was 510 days (692.30 ± 626.72) in the ENZ group and 180 days (495.90 ± 498.51) in the CBZ group. The CBZ group was younger than the ENZ group, and the CBZ group received fewer cycles of DOC and had a shorter time to CRPC than the ENZ group. The CBZ group was expected to include more high-risk patients than the ENZ group; however, the ENZ group had a higher proportion of patients with bone metastasis than the CBZ group in this study. (ENZ vs. CBZ group: 43.9% vs. 42.0%). The TROPIC study compared CBZ with ENZ and ABI after DOC in patients with mCRPC. The CARD trial compared CBZ with ENZ and ABI after DOC in mCRPC. The CARD trial compared CBZ with ABI or ENZ in 225 patients with CRPC who had previously been treated with DOC for mCRPC and who subsequently progressed within 12 months on ABI or ENZ. The results showed that CBZ and ARSI were associated with an OS benefit of 13.6 months and 11.0 months, respectively (HR = 0.64) [15]. It is speculated that this is due to the fact that CBZ maintains its activity while ARSI allows cross-resistance [16]. In the present study, the OS of patients treated with ENZ and ABI was comparable to the previous studies, while the median OS of patients who received CBZ in the present study was shorter, at 258 days (mean: 364.80 ± 472.77). The phase III TROPIC trial compared mCRPC treated with CBZ and mitoxantrone after DOC in 755 patients (CBZ group, n = 378; mitoxantrone group, n = 377) [17]. In that study, the OS of the CBZ group was 15.1 months, indicating the benefit of CBZ after DOC treatment (HR, 0.70). Although the CARD and TROPIC trials used DOC for CRPC and therefore cannot simply be compared with this study, it is expected that the number of patients who were eligible for chemotherapy after upfront DOC in Japan was higher than the number of patients who were treated with ARSI. It was expected that there would be more high-volume cases than cases in which ARSI was used in Japan. Based on these reports, ENZ is currently the preferred drug for use after upfront DOC. However, it may be necessary to re-examine this issue when the use of CBZ in low-volume patients increases in Japan in the future.

In this study, there was no significant difference in the OS between the groups who received ENZ and ABI as the second drug after upfront DOC for mCSPC (HR = 0.94). The OS of the two groups using ABI and ENZ as the third-line drug was further analyzed. There was no difference in OS between the ENZ–ABI and ABI–ENZ groups (ENZ–ABI vs. ABI–ENZ, *p* = 0.85; HR, 1.03) (Figure The efficacy of ABI after DOC for mCRPC was demonstrated in the COU-AA-301 trial, which randomized 1195 patients with mCRPC previously treated with DOC, with 797 in the ABI group, and 398 in the placebo group. The progression-free survival (PFS) was 5.6 months in the ABI group and 3.6 months in the placebo group (*p* < 0.001), while OS was 14.8 months in the ABI group and 10.9 months in the placebo group, both of which amounted to significant differences (*p* < 0.001) [18]. Nooman et al. [19] and Loriot et al. [20] analyzed the cross-resistance to ENZ followed by ABI in a retrospective study of PFS and OS in a real clinical setting. They reported that 18 patients (60% of all patients) had a PSA reduction of ≥50% after ENZ, whereas only 1 out of 27 patients (4%) had a PSA reduction of ≥50% or more after ENZ followed by ABI. The median PFS was 15.4 weeks (95% CI 12.4–21.3) and OS was 50.1 weeks (95% CI 28.3–72.0) in patients who received ENZ followed by ABI [19].

Loriot et al. also performed a retrospective analysis of 38 patients with mCRPC who were pretreated with DOC, followed by ENZ and ABI. Twenty-one patients (55% of all patients) had a ≥50% PSA reduction after ENZ, while three patients (8% of all patients) who subsequently received ABI had a ≥50% PSA reduction (95% CI 2% to 21%). The median PFS of patients who received ENZ followed by ABI was 2.7 months (95% CI 2.3–4.1), while OS was 7.2 months (95% CI 7.3-not reached) [20]. A possible mechanism of cross-resistance between ABI, a CYP17 inhibitor, and ENZ, an androgen receptor (AR) inhibitor, was reported by [21], who suggested that the formation of receptor mutants lacking the ligand-binding domain in the AR may lead to homeostatic activation of the AR in the absence of androgen. The usefulness of ABI after DOC in the COU-AA-301 trial, and the cross-resistance between ABI and ENZ, were reported by Loriot et al. [19] and Nooman et al. [20], who considered ABI to be the best drug and ENZ to be the second-best drug for administration after DOC. In contrast, the present study found that the OS of mCSPC patients who were treated upfront with DOC did not differ between those who received ABI and those who received ENZ as the next-line therapy. After upfront DOC, ENZ or ABI was considered a good choice. These results provide an option for patients who cannot use either drug owing to side effects or when either drug is not available, including cases in which it is not available for financial reasons.

The present study had several limitations. First, it was a retrospective study based on an analysis of an insurance database, and there was little patient information including the stage, presence of visceral metastasis, pathological information, and performance status. Although some selection bias was introduced by the retrospective nature of the database study, this study was conducted on a large scale with 25,936 participants. Second, the dosage of the therapeutic drug and the reason for discontinuation were not listed in the database. It is therefore unclear whether the treatment was discontinued because of ineffectiveness or side effects.

## 5. Conclusions

Enzalutamide prolonged OS in comparison to CBZ after upfront DOC. In addition, the duration of drug treatment after upfront DOC was prolonged in patients who received ENZ in comparison to those who received ABI or CBZ. Future research is needed to determine the mechanistic differences among the three drugs (ENZ, ABI, and CBZ) that are responsible for this result.

## Figures and Tables

**Table 1:** Patient characteristics.

Number (%) Median (Mean ± SD)						
	ENZ	ABI	CBZ	ENZ vs. ABI	ENZ vs. CBZ	ABI vs. CBZ
**Number of patients**	**998**	**617**	**382**			
**Age (yrs)**	**73 (72.9 ± 6.98)**	**73 (72.6 ± 7.09)**	**72 (71.1 ± 7.36)**	***p* = 0.50**	***p* < 0.0001**	***p* = 0.001**
**18–64 years**	**97 (9.7%)**	**76 (12.32%)**	**62 (16.2%)**			
**65–74 years**	**467 (46.8%)**	**276 (44.73%)**	**188 (49.2%)**			
**75–88 years**	**428 (42.9%)**	**264 (42.8%)**	**131 (34.3%)**			
**≥** **89 years**	**5 (1.1%)**	**1 (0.2%)**	**1 (0.3%)**			
**Number of DOC**	**8 (12.4 ± 13.23)**	**8 (11.9 ± 12.71)**	**6 (8.6 ± 8.39)**	***p* = 0.35**	***p* < 0.0001**	***p* = 0.0003**
**time to CRPC (days)**	**510 (692.3 ± 626.72)**	**420 (583.6 ± 569.79)**	**180 (495.9 ± 498.51)**	***p* = 0.0002**	***p* < 0.0001**	***p* < 0.0001**

ENZ: enzalutamide; ABI: abiraterone acetate; CBZ: cabazitaxel.

## Data Availability

The raw data to create tables and figures are available upon request.

## Supplementary Materials

The following supporting information can be downloaded at: https://www.mdpi.com/article/10.3390/diseases12070162/s1, Figure S1: Kaplan Meier Curve in second-line treatment (enzalutamide or abiraterone acetate). No significant difference in overall survival was obtained between the enzalutamide and abiraterone acetate groups (*p* = 0.54, HR 0.94).
