# Supplementary material for: Enzalutamide Prolonged the Duration of Drug Use in Comparison to Abiraterone Acetate and Cabazitaxel after Upfront Docetaxel: A Large Japanese Database Study
Source: Diseases. 2024 Jul 18;12(7):162. doi: 10.3390/diseases12070162 (PMC11276074; doi:10.3390/diseases12070162)
Supplement: Supplementary file 1 [file diseases-12-00162-s001.zip › diseases-3060955-supplementary.pdf]

Supplementary Fig.1

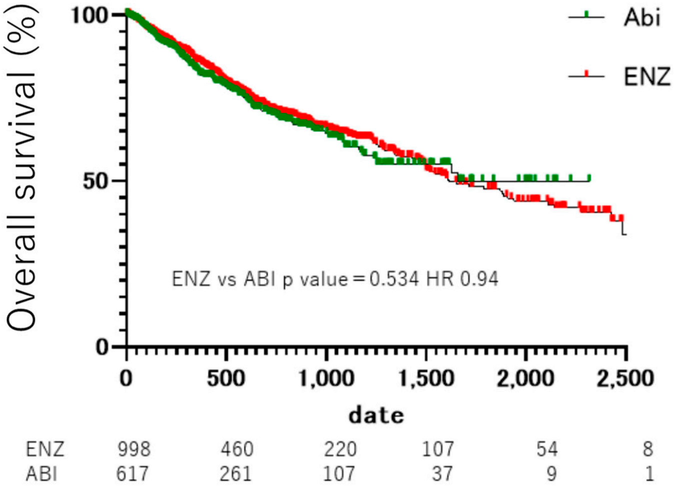

ENZ: enzalutamide, ABI: abiraterone acetate

Figure S1. Kaplan Meier Curve in second line treatment (enzalutamide or abiraterone acetate). No significant difference in overall survival was obtained between the enzalutamide and abiraterone acetate groups (p = 0.54, HR 0.94).
